# Impact of intention and feeling toward being pregnant on postpartum depression: the Japan Environment and Children’s Study (JECS)

**DOI:** 10.1007/s00737-018-0938-7

**Published:** 2018-12-27

**Authors:** Sachiko Baba, Takashi Kimura, Satoyo Ikehara, Kaori Honjo, Ehab S. Eshak, Takuyo Sato, Hiroyasu Iso, Japan Environment & Children’s Study Group, Toshihiko Kawamoto, Hirohisa Saito, Reiko Kishi, Nobuo Yaegashi, Koichi Hashimoto, Chisato Mori, Shuichi Ito, Zentaro Yamagata, Hidekuni Inadera, Michihiro Kamijima, Takeo Nakayama, Hiroyasu Iso, Masayuki Shima, Yasuaki Hirooka, Narufumi Suganuma, Koichi Kusuhara, Takahiko Katoh

**Affiliations:** 1 Bioethics and Public Policy, Department of Social Medicine, Osaka University Graduate School of Medicine, Yamadaoka 2-2, Suita, Osaka 565-0871 Japan; 2 Center for International Relations, Osaka University Graduate School of Medicine, Yamadaoka 2-2, Suita, Osaka 565-0871 Japan; 3 Public Health, Department of Social Medicine, Osaka University Graduate School of Medicine, Yamadaoka 2-2, Suita, Osaka 565-0871 Japan; 4 Department of Hygiene and Public Health, Osaka Medical College, 2-7 Daigakumachi, Takatsuki-shi, Osaka 569-8686 Japan; 5 Psychology and Behavioral Sciences, Faculty of Medicine, Faculty of Medicine, Osaka Medical College, Daigaku-machi 2-7, Takatsuki, Osaka 569-8686 Japan; 6 Osaka Women’s and Children’s Hospital, 840 Murodo cho, Izumi, 594-1101 Japan

**Keywords:** Unplanned pregnancy, Unwanted pregnancy, Emotion, Happiness, Postpartum depression

## Abstract

Pregnancy intention is reported to be associated with the risk of postpartum depression (PPD), but the impact of feelings toward being pregnant on PPD is unknown. We aimed to examine whether feelings toward being pregnant are associated with PPD at 1 month after childbirth. In our nationwide study between 2011 and 2014 in Japan, we used multivariate logistic regression analyses to examine the associations between pregnancy intention and feelings toward being pregnant with PPD [Edinburgh Postnatal Depression Scale (EPDS score > 9 or > 12)] among Japanese women. Among 92,431 women, 14.0 and 5.4% had PPD with EPDS scores > 9 and > 12, respectively. Compared with women who felt very happy to be pregnant, those whose pregnancy was unintended but happy, unintended and confused, those who felt troubled, and those who felt no emotion toward being pregnant had increased risks of PPD [multivariable odds ratios (95% confidence intervals (CIs)) = 1.17 (1.11–1.22), 1.39 (1.29–1.49), 1.74 (1.42–2.14), and 1.58 (1.22–2.02), respectively, for EPDS score > 9]. Those associations were more evident without antenatal possible mental illness (K6 score < 13). Women whose pregnancy was unintended should be regarded as targets for the early detection and prevention of PPD irrespective of whether they felt happy or confused.

## Introduction

Postpartum depression (PPD) is a major concern in reproductive health (O’Hara and McCabe 2013). The prevalence of PPD differs by the diagnosis criteria and timing (O’Hara and Swain 1996) but is reported to be between 9 and 24% among Japanese women (Ministry of Health, Labour, and Welfare 2013; Norhayati et al. 2015) and between 10 and 15% among US women (Patel et al. 2012). Recently, the time period of diagnosis was stated in the Diagnostic and Statistical Manual of Mental Disorders 5th edition (DSM-5), and PPD is defined as depressive episodes that occur during pregnancy and within 4 weeks of childbirth (American Psychiatric Association 2013). Thus, monitoring and evaluating the prevalence and risk factors for PPD according to the defined period are essential. PPD is associated with adverse health outcomes for infants (Stein et al. 1991; Kingston et al. 2012) as well as for mothers (van Wijngaarden et al. 2004; Pearlstein et al. 2009); therefore, efforts to detect and decrease the prevalence of PPD have been made (Ministry of Health, Labour, and Welfare 2013; Ko et al. 2017). In Japan, the national and municipal government began financially supporting postpartum assessment for PPD screening at 2 weeks and 1 month after childbirth (Japan Association of Obstetricians and Gynecologists 2017).

On the other hand, half of all pregnancies have been reported as unintended in both low- and high-income countries (Sedgh et al. 2014; Finer and Zolna 2016). Previous studies have shown the association of unintended pregnancies with PPD. A multi-center study conducted with 290 Japanese women reported that women not desiring to be pregnant had a higher proportion of major depressive episodes during pregnancy and at three postpartum months (Kitamura et al. 2006); however, they did not examine the association between the desire for pregnancy and major depressive episodes probably because of the small number of participants. A hospital-based study conducted with 2076 Korean women reported the association of unintended pregnancy with the risk of PPD at 1, 4, 12, and 24 postpartum months, but they did not include information on the antenatal possible mental illness of the studied women (Bahk et al. 2015). Another hospital-based study conducted with 688 US women has revealed the association of unintended pregnancy with the risk of PPD at 3 and 12 postpartum months (Mercier et al. 2013). However, besides its moderate sample size, pregnancy intention was examined during the second trimester in that study, which might have led to a recall bias.

According to the literatures mentioned above, unintended pregnancy is a risk factor for PPD; however, not all unintended pregnancies are regarded as unhappy. In a study conducted on pregnant women in the USA, half of the unintended pregnancies were perceived as happy (Sable and Libbus 2000). There might be ambiguous feelings toward being pregnant. Therefore, it might be difficult to dichotomously express feelings (intended or unintended, happy or unhappy), and combined categories of feelings may be useful to reflect the actual emotional status of pregnant women. To the best of our knowledge, no study has evaluated the association of feelings toward being pregnant with PPD at one postpartum month with a large sample size.

The Japan Environment and Children’s Study (JECS) assessed information about feelings toward being pregnant, including pregnancy intention and PPD at 1 month after childbirth using large nationwide population samples. Using these data, we compared the risk of PPD among women who felt very happy to be pregnant, and those whose pregnancy was unintended but felt happy, unintended and felt confused, felt troubled, and those who felt no emotion toward being pregnant.

## Materials and methods

### Study population

The JECS is a nationwide government-funded birth cohort study that began recruiting expectant mothers in January 2011. Fifteen Regional Centers (Hokkaido, Miyagi, Fukushima, Chiba, Kanagawa, Koshin, Toyama, Aichi, Kyoto, Osaka, Hyogo, Tottori, Kochi, Fukuoka, and South Kyushu/Okinawa) were selected. Women were recruited during early pregnancy at obstetrics clinics/hospitals and/or local municipal offices issuing mother and child health handbooks. Recruitment started in January 2011 and continued until March 2014. The JECS protocol has been published elsewhere (Kawamoto et al. 2014; Michikawa et al. 2018). The details of the JECS have been described in previous studies (Michikawa et al. 2015; Suzuki et al. 2016). The study included data measured at enrollment during the first trimester, at pregnancy checkup examination during the second trimester, at delivery, and at postpartum 1 month by pregnant women and obstetricians.

### Participants

Among 103,099 pregnancies, we only included those with a live singleton infant (*n* = 98,259). The complete information on pregnancy intention and their feelings toward being pregnant, and PPD status was available for 92,431 pregnancies out of the 98,259 recruited pregnancies (94.1%).

### Ethical issues

The JECS protocol was reviewed and approved by the Ministry of the Environment’s Institutional Review Board on Epidemiological Studies and by the Ethics Committees of all participating institutions. The JECS was conducted in accordance with the Declaration of Helsinki and other nationally valid regulations and guidelines.

### Variables

Data on women’s feelings toward being pregnant, including pregnancy intention, were collected at the time of the registration generally during the first trimester. Data were primarily combined and categorized into five groups according to the women’s answers to the question “When you recognized the current pregnancy, how did you feel?” (1) Very happy, (2) unintended but happy, (3) unintended and confused, (4) troubled, and (5) no emotion.

We collected a Japanese-language Edinburgh Postnatal Depression Scale (EPDS) score at 1 month after childbirth. EPDS (Cox et al. 1987), which comprises 10 items listed in a four-point Likert scale from 0 to 3 according to the increasing severity of symptoms, ranges from “Yes, most of the time,” “Yes, sometimes,” “Not very often,” to “No, not at all.” Okano et al. translated the EPDS into Japanese, back-translated the scale, conducted the test–retest method to examine reliability, and calculated Cronbach’s α (0.78) (Okano et al. 1996). Okano et al. reported that the appropriate cutoff point for detecting PPD was 9 or higher, with 75% sensitivity and 93% specificity (Yamaoka et al. 2016; Muchanga et al. 2017), whereas 12 or higher is generally used as the cutoff point worldwide (Cox et al. 1987). A recent study on Japanese women has shown the optimal cutoff of EPDS score for major depressive episode during pregnancy as 12 (Usuda et al. 2017). We, therefore, used both cutoff points > 9, and > 12, in this study.

The covariates included women’s age, parity, marital status, smoking habit, education, annual family equivalent income, intimate partner verbal violence at pregnancy (IPV), possible mental illness during a month prior to enrollment, history of antidepressant use for the past 1 year, and residential area. Marital status, smoking habit, IPV, and antenatal possible mental illness were obtained using the medical records at the registration; education and income during the second trimester; parity at delivery from the obstetrician, and age were obtained from the obstetrician’s record 1 month after childbirth. The information in the medical records was transcribed by physicians, midwives/nurses, or research coordinators. Women’s age was categorized as 5-year age groups; parity as primiparous or multiparous; marital status as married, never (not yet), divorced, and widowed; smoking as never smokers, former smokers who previously smoked but quit before pregnancy, quit smokers who stopped smoking during pregnancy, and continued smokers who continued to smoke during pregnancy; education as junior high school, high school, vocational school, junior college, and university and higher; and IPV as none, rarely, sometimes, and often. Equivalent income was calculated by dividing the average amount in 6-scale family income categories by the square root of the number of family members and re-categorized into quartiles.

Women’s possible mental illness during the past month prior to enrollment was assessed using a Japanese version of the Kessler 6 (K6) scale. The K6 score is originally used for screening “serious mental illness” (Kessler et al. 2003) or mood and anxiety disorders (Furukawa et al. 2003) in the general population, with a cutoff point of 13 or higher (Kessler et al. 2003; Furukawa et al. 2003). A Japanese version of K6 was developed and validated with a similar cutoff point (12/13) (Furukawa et al. 2008) Therefore, we regarded K6 score > 13 as possible mental illness. The history of antidepressant use was determined by a question regarding the use of antidepressants in the past year. Residential areas were represented by the locations of the 15 Regional Centers where the data were collected.

### Statistical analyses

Multivariable logistic regression analyses were used to estimate the association between feelings toward being pregnant and the risk of PPD. Odds ratios (ORs) and 95% confidence intervals (CIs) were calculated after adjusting for potential confounders. Using the category of women with a “very happy” feeling as the reference group, we estimated the risk of PPD (EPDS score > 9 and > 12) among women whose pregnancy was unintended but happy, unintended and confused, felt troubled, and those who had no emotion toward being pregnant. We also used multivariable logistic regression analyses stratified by women’s antenatal possible mental illness to examine whether the antenatal possible mental illness modifies the association between the feelings toward being pregnant and PPD (EPDS score > 9 and > 12). All analyses were performed using Statistical Analysis Software version 9.4 (SAS Institute, Inc., Cary, NC, USA).

## Results

Table 1 shows the characteristics of 92,431 women. Among them, 14.0 and 5.4% had PPD with EPDS scores > 9 and > 12, respectively. Women who regarded their pregnancy as unintended and confused, felt troubled, and those who felt no emotion toward being pregnant had higher proportions of PPD than those who regarded their pregnancy as very happy. High proportions of PPD were also seen in younger, in primiparous, in never married, divorced, or widowed women, in women who quit or continued smoking when pregnant, in women with lower education, in women who experienced IPV, in women with a mental illness (K6 score > 13), and in women with a history of antidepressant use.

**Table 1:** Characteristics of participants

Characteristics		Number of women	Postpartum depression (EPDS ≥ 9)		Postpartum depression (EPDS ≥ 12)	
		*n*	*n*	Proportion (%)	*n*	Proportion (%)
	Total	92,431	12,943	14.0	4951	5.4
Pregnancy intention and feeling of being pregnant						
	Very happy	61,045	7369	12.1	2584	4.2
	Unintended but happy	23,997	3879	16.2	1598	6.7
	Unintended and confused	6425	1411	22.0	613	9.5
	Troubled	535	186	34.8	112	20.9
	No emotion	429	98	22.8	44	10.3
Age, year	< 20	737	181	24.6	84	11.4
	20–29	33,772	5507	16.3	2205	6.5
	30–39	53,737	6720	12.5	2442	4.5
	40+	4185	535	12.8	220	5.3
Parity	Primiparous	36,259	6505	17.9	2502	6.9
	Multiparous	54,048	6054	11.2	2308	4.3
	Missing	2124	384	18.1	141	6.6
Marital status	Married	88,186	11,900	13.5	4463	5.1
	Never (not yet)	3126	738	23.6	321	10.3
	Divorced	734	206	28.1	114	15.5
	Widowed	15	4	26.7	2	13.3
	Missing	370	95	25.7	51	13.8
Smoking	Never	53,721	6,623	12.3	2428	4.5
	Former	21,795	3,021	13.9	1107	5.1
	Quit smoking	12,028	2248	18.7	939	7.8
	Continued smoking	4254	934	22.0	429	10.1
	Missing	633	117	18.5	48	7.6
Education	Junior high school	4233	940	22.2	457	10.8
	High school	28,682	4681	16.3	1929	6.7
	Vocational school	22,509	2961	13.2	1060	4.7
	Junior college	16,155	1961	12.1	668	4.1
	University and higher	19,907	2243	11.3	757	3.8
	Missing	945	157	16.6	80	8.5
Family income (equivalent income)						
	Q1 (lowest)	24,921	3895	15.6	1616	6.5
	Q2	13,069	2279	17.4	890	6.8
	Q3	28,508	3513	12.3	1236	4.3
	Q4 (highest)	18,855	2034	10.8	686	3.6
	Missing	7078	1222	17.3	523	7.4
Intimate partner violence						
	None	80,857	9934	12.3	3563	4.4
	Rarely	7461	1761	23.6	776	10.4
	Sometimes	3128	906	29.0	430	13.7
	Often	689	274	39.8	143	20.8
	Missing	296	68	23.0	39	13.2
Antenatal possible mental illness (K6 score)						
	No (K6 < 13)	89,306	11,211	12.6	3905	4.4
	Yes (K6 > 13)	3125	1732	55.4	1046	33.5
History of antidepressant use						
	No	87,755	11,291	12.9	4072	4.6
	Yes	4676	1652	35.3	879	18.8

Compared with women who felt very happy to be pregnant, women who felt their pregnancy was unintended but happy, unintended and confused, or those who felt no emotion toward being pregnant had an increased risk of PPD: OR (95% CI) = 1.25 (1.20–1.31), 1.64 (1.53–1.76), and 1.67 (1.30–2.13), respectively, for EPDS score ≥ 9, and OR (95% CI) = 1.36 (1.27–1.46), 1.67 (1.51–1.85), and 1.78 (1.26–2.50), respectively, for EPDS score ≥ 12 (Table 2). Women who felt their pregnancy was troubled had the largest risk of PPD compared with those with other feelings: OR (95% CI) = 2.25 (1.83–2.75) for EPDS score ≥ 9 and 2.85 (2.23–3.64) for EPDS score ≥ 12.

**Table 2:** Multivariate odds ratios (ORs) of postpartum depression (PPD) according to feeling toward being pregnant

	PPD (EPDS score > 9)	PPD (EPDS score > 12)
	Multivariable^a^ OR (95% CI)	Multivariable^a^ OR (95% CI)
Very happy	Ref	Ref
Unintended but happy	1.25 (1.20–1.31)	1.36 (1.27–1.46)
Unintended and confused	1.64 (1.53–1.76)	1.67 (1.51–1.85)
Troubled	2.25 (1.83–2.75)	2.85 (2.23–3.64)
No emotion	1.67 (1.30–2.13)	1.78 (1.26–2.50)

^a^Adjusted for mother’ age, parity, marital status, smoking, mother’s education, household income, intimate partner violence, antenatal possible mental illness (K6), history of antidepressant use, and residential area

The associations between feelings toward being pregnant and the risk of PPD stratified by antenatal possible mental illness are shown in Table 3. Those associations were generally stronger among women without possible mental illness (K6 score < 13). In those with possible mental illness (K6 score > 13), increased risks for PPD were observed only among women who felt their pregnancy as unintended and confused.

**Table 3:** Adjusted odds ratios of postpartum depression (PPD) according to feeling toward being pregnant, stratified by antenatal possible mental illness (K6 score)

		PPD (EPDS score ≥ 9)		PPD (EPDS score ≥ 12)	
	No. at risk	No. of PPD	Multivariable^a^ OR (95% CI)	No. of PPD	Multivariable^a^ OR (95% CI)
No antenatal possible mental illness (K6 score < 13) (*n* = 89,306)					
Very happy	59,564	6608	Ref	2153	Ref
Unintended but happy	23,017	3321	1.26 (1.20–1.32)	1257	1.38 (1.28–1.49)
Unintended and confused	5905	1081	1.65 (1.54–1.78)	404	1.69 (1.51–1.90)
Troubled	429	120	2.55 (2.04–3.18)	60	3.21 (2.41–4.29)
No emotion	391	81	1.85 (1.43–2.38)	31	1.87 (1.28–2.74)
Antenatal possible mental illness (K6 score ≥ 13) (*n* = 3125)					
Very happy	1481	761	Ref	431	Ref
Unintended but happy	980	558	1.13 (0.95–1.34)	341	1.19 (0.99–1.43)
Unintended and confused	520	330	1.44 (1.16–1.79)	209	1.47 (1.18–1.83)
Troubled	106	66	1.25 (0.81–1.91)	52	1.97 (1.30–3.00)
No emotion	38	17	0.74 (0.37–1.47)	13	1.24 (0.60–2.55)

^a^Adjusted for mother’ age, parity, marital status, smoking, mother’s education, household income, intimate partner violence, history of antidepressant use, and residential area

## Discussion and conclusions

In this nationwide study, we found that women who felt their pregnancy was unintended but happy, those who felt their pregnancy was unintended and confused, and those who felt no emotion toward being pregnant had increased risks of PPD (EPDS score > 9 and > 12) after adjusting for demographic, socioeconomic, and antenatal mental factors. We also found that women who felt troubled with their pregnancy had the highest risk of PPD. The risks of PPD with EPDS score > 12 were generally higher than those of PPD with EPDS score > 9.

Our result that unintended pregnancy was associated with increased risk of PPD was consistent with previous findings in Japan and other high-income countries (Mercier et al. 2013; Kitamura et al. 2006; O’Hara and McCabe 2013; Bahk et al. 2015; Beck 2001).

In the present study, we unexpectedly found an increased risk of PPD among women who felt their pregnancy was unintended but happy. There was a previous report in African-American and Latin populations stating that pregnancy intentions and happiness were strongly associated, but happiness was a better predictor of reduced PPD rather than intention (Blake et al. 2007). Our finding did not meet this notion possibly due to differences in ethnicity, culture, and participants’ characteristics.

In the analysis stratified by antenatal possible mental illness, the associations were weaker among women with possible mental illness, which implies that antenatal possible mental illness modified the association between their feelings toward being pregnant and risk of PPD.

The biological mechanism underlying PPD remains unclear (Patel et al. 2012). Changes in the levels of several hormones after delivery have been suggested to induce PPD, but the findings were inconsistent (Soares and Zitek 2008; Patel et al. 2012; O’Hara and McCabe 2013). In contrast, regarding psychological mechanisms, the feeling toward being pregnant plays a role in PPD; in addition to history of depression and antenatal possible mental illness, antenatal anxiety, perinatal stress, feeling pessimistic during the antenatal period, and fear of childbirth have been considered to be predictors of PPD (O’Hara et al. 1991; Kitamura et al. 2006; Beck 2001, Condon and Watson 1987; Räisänen et al. 2013). Regarding the mechanism by which negative feelings or antenatal possible mental illnesses may affect the risk of PPD, the bonding of mother to fetus may play a role. An association between anxiety during pregnancy and risk of PPD was shown to be mediated by bonding failure (Kokubu et al. 2012). Considering mother-to-infant bonding, which comprises two factors; “anger and rejection” and “lack of affection” (Cattell 1966; Taylor et al. 2005), feeling troubled by being pregnant may correspond to “anger and rejection” component, and feeling no emotion may correspond to “lack of affection” component. Bonding failure between mother and fetus may persist after childbirth.

The major strengths of this study include a cohort study with a large sample size, in which the information on pregnancy intention and antenatal possible mental illness was collected during early pregnancy, precluding the possibility of recall bias. We adjusted for potential confounding variables such as maternal demographic characteristics, socioeconomic factors, and antenatal possible mental illness. Another strength is that the outcome was shown as both PPD with EPDS scores > 9 and > 12, and was evaluated during postpartum 1 month. The findings of this study may be useful to prevent and control PPD through the early detection of high-risk individuals. Antenatal screening for PPD through a questionnaire on feelings toward being pregnant is possible using antenatal regular check-ups at obstetrics clinics/hospitals or at local municipal offices issuing mother and child health handbooks.

This study is one of the few studies that demonstrate the association between feelings toward being pregnant and risk of PPD. However, several limitations should be addressed. First, the intention and feelings were not assessed separately. The questionnaire had missed some combinations such as unintended but very happy pregnancies, and intended but troubled pregnancies. Second, the questionnaire on pregnancy intention and feelings toward being pregnant was not validated. However, it is logically difficult because of no measurement golden standards for the comparison. Third, our measurements, EPDS and K6, are self-reported and not diagnosed by psychiatrists. However, EPDS and K6 are commonly used in research and are well-validated, reliable measurement; therefore, the impact of this error is expected to be small. Fourth, residual confounding could have occurred from unmeasured confounding variables, such as mothers’ chronic diseases.

In conclusion, our findings show that women whose pregnancy was unintended but felt happy had an increased risk of PPD. Women who regarded their pregnancies as unintended and felt confused, felt troubled, or had no specific emotion were also associated with an increased risk of PPD. These high-risk women should be regarded as targets for the early detection and prevention of PPD.
